# Inflammation in Coronary Microvascular Dysfunction

**DOI:** 10.3390/ijms222413471

**Published:** 2021-12-15

**Authors:** Marios Sagris, Panagiotis Theofilis, Alexios S. Antonopoulos, Evangelos Oikonomou, Christina Paschaliori, Nikolaos Galiatsatos, Kostas Tsioufis, Dimitris Tousoulis

**Affiliations:** 1 Cardiology Clinic, ‘Hippokration’ General Hospital, School of Medicine, National and Kapodistrian University of Athens, 11527 Athens, Greece; panos.theofilis@hotmail.com (P.T.); alexios.antonopoulos@cardiov.ox.ac.uk (A.S.A.); boikono@gmail.com (E.O.); cpasxali@gmail.com (C.P.); nikosgaliatsatos1957@gmail.com (N.G.); kptsioufis@gmail.com (K.T.); drtousoulis@hotmail.com (D.T.); 2 Department of Cardiology, “Sotiria” Thoracic Diseases Hospital of Athens, University of Athens Medical School, 11527 Athens, Greece

**Keywords:** coronary microvascular dysfunction, atherosclerosis, imaging, biomarkers, risk factors, anti-inflammatory treatment

## Abstract

Chronic low-grade inflammation is involved in coronary atherosclerosis, presenting multiple clinical manifestations ranging from asymptomatic to stable angina, acute coronary syndrome, heart failure and sudden cardiac death. Coronary microvasculature consists of vessels with a diameter less than 500 μm, whose potential structural and functional abnormalities can lead to inappropriate dilatation and an inability to meet the required myocardium oxygen demands. This review focuses on the pathogenesis of coronary microvascular dysfunction and the capability of non-invasive screening methods to detect the phenomenon. Anti-inflammatory agents, such as statins and immunomodulators, including anakinra, tocilizumab, and tumor necrosis factor-alpha inhibitors, have been assessed recently and may constitute additional or alternative treatment approaches to reduce cardiovascular events in atherosclerotic heart disease characterized by coronary microvascular dysfunction.

## 1. Introduction

Ischemic cardiac pain in the context of non-obstructed epicardial coronary arteries, a phenomenon frequently encountered in clinical cardiology practice, has been recognized as a clinical entity of increased cardiovascular risk when compared to control subjects [1,2], contrary to initial theories suggesting a nonthreatening disease progression [3]. Coronary microvascular dysfunction (CMVD) consists of the main etiologic factor of peripheral ischemia with “normal” epicardial coronary arteries. However, no definitive data exist on the pathophysiology, diagnosis and treatment of CMVD.

Coronary microvasculature consists of vessels with a diameter less than 500 μm, whose remodeling due to various stimuli could lead to structural and functional abnormalities and, consequently, result in inappropriate dilatation and the inability to meet the required oxygen demands. Known cardiovascular risk factors, such as diabetes mellitus and arterial hypertension, are implicated in this process due to their deleterious vascular effects. Ultimately, patients with CMVD could progress to a phenotype of heart failure with preserved ejection fraction, with variable prognosis due to a lack of disease-specific treatment [4].

Chronic low-grade inflammation is undoubtedly involved in coronary atherosclerosis. Importantly, favorable cardiovascular outcomes have been reported in recent trials of patients with documented coronary artery disease (CAD) receiving either broad based or target anti-inflammatory treatment [5,6,7]. Contemporary evidence suggests that an inflammatory background is also responsible for the development of CMVD [8]. Therefore, we review the latest data regarding CMVD epidemiology and diagnostic approach while elaborating on the speculated effects of inflammation and the potential therapeutic implications of immunomodulatory agents.

## 2. Multimodality Assessment of CMVD

Evaluation of coronary blood flow velocity and coronary blood flow after the administration of vasodilating substances during cardiac catheterization are important determinants of coronary flow reserve (CFR), a measure of epicardial and microcirculatory blood flow. In the absence of epicardial artery obstruction, a reduced CFR value is indicative of CMVD. Corrections for age and systolic blood pressure in individuals should always be implemented. However, scientific research is currently focused on noninvasive methods of CMVD assessment.

### 2.1. Echocardiography

To begin with, echocardiography has been involved in CMVD assessment since 1998, when Wei et al. infused air-filled albumin microbubbles intravenously in an effort to quantify myocardial blood flow (MBF) in an epicardial coronary artery. Next generation microbubbles, destructible by ultrasound, allowed the more accurate calculation of MBF by assessing their mean velocity (rate of reappearance after destruction in the setting of a constant intravenous infusion) and the microvascular cross-sectional area (microbubble concentration in the myocardium) [9]. Apart from contrast echocardiography, Doppler echocardiographic assessment of coronary flow velocity reserve (ratio of coronary flow velocity at stress and rest at proximal left anterior descending coronary artery) was found to satisfactorily correspond with invasive methods [10,11]. Moreover, its prognostic efficacy was determined in high risk individuals with known or suspected CAD [12]. Even though echocardiography is attractive due to the nature of the procedure (bedside, inexpensive, minimal patient-related risk), it is operator-dependent and has not been validated while patient-related factors such as obesity or pulmonary pathology could hinder the quality of measurements.

### 2.2. Cardiac CT Angiography

Cardiac CT angiography (CCTA) appears to be another appealing approach in the assessment of CMVD, since information about coronary anatomy and myocardial perfusion can be acquired from one study [13]. By obtaining electrocardiographically-gated CT perfusion images at rest and after stress with vasodilators, MBF can be quantified [14]. Moreover, fractional flow reserve and MBF can also be calculated by mathematical models simulating maximal hyperemia [15]. However, the increased radiation exposure paired with a higher risk of contrast-induced acute kidney injury especially in individuals with preexisting renal disease should always be taken into account [16].

### 2.3. Fat Attenuation Index

Interestingly, recent advancements in the detection of coronary inflammation via perivascular fat imaging in CT has garnered scientific attention and could be also applied in the context of CMVD [17]. The fat attenuation index (FAI), a recently developed imaging marker, was increased in areas of profound atherosclerosis as well as in regions characterized by fractional flow reserve (FFR) of 0.75 or less [18,19], highlighting the existing burden of inflammation, which is a distinct characteristic of vulnerable plaques. It has to be noted that FAI values appear to be correlated with the gold standard method of perivascular inflammation imaging, PET-CT with 18F-NaF uptake, FAI values seemed to be correlated with 18F-NaF uptake, as shown in a recent small cohort study of 41 stable patients with high risk plaques [20]. Evaluation of the pericoronary adipose tissue may be of additive discriminative importance in highly stenotic atherosclerotic plaques and improve their evaluation by CCTA, since a higher FAI value is indicative of a more hemodynamically significant stenosis.

FAI may also be of use in the setting of an acute myocardial infarction, with higher FAI values around the culprit lesions compared to FAI around non-culprit lesions. At the follow-up evaluation, the FAI around the culprit lesion was lower than baseline, with values comparable to those detected around stable atherosclerotic regions [17,21,22]. According to this observation, the capability of FAI to detect the acute changes in the inflammatory burden of pericoronary fat is evident, with a good discrimination ability (AUROC = 0.70) [21]. The fat radiomic profile (FRP) of stable pericoronary fat changes in comparison to follow-up CCTA imaging has also been described by Oikonomou et al. [23]. Several pharmacologic interventions could be implicated in the changes of pericoronary fat detected by FAI including drugs such as aspirin, statins, or biologic therapy with anti-inflammatory agents [24].

The incremental prognostic value of perivascular adipose tissue attenuation could become an important imaging option towards further characterization of the residual cardiovascular risk, which may not be adequately estimated by traditional scoring methods or inflammatory biomarkers. It is believed that the presence of residual inflammation in the coronary arterial bed and its detection might aid towards a more precise risk stratification and, consequently, the institution of an appropriate treatment approach, which might include anti-inflammatory agents. The use of statin and aspirin might modify this residual risk and, in such cases, FAI is no longer predictive of incident cardiovascular risk [24]. However, FRP and its properties constitute a non-modifiable cardiovascular risk factor, not influenced by drug therapy [23].

FAI, other than being an attractive alternative imaging option regarding the detection of vascular inflammation, may also be less expensive, with lower radiation exposure for the patient. However, its association with the existence of CMVD has not been explored and further research is required in this direction to better define FAI’s role in the evaluation of CMVD.

### 2.4. Cardiac Positron Emission Tomography

The most extensively studied noninvasive method of MBF estimation has undoubtedly been cardiac Positron Emission Tomography (PET) [25]. Even though limitations exist regarding its cost and availability of cyclotrons, myocardial perfusion reserve has been associated with adverse outcomes, namely heart failure with preserved ejection fraction, on top of its role in diagnosing CMVD [26,27].

### 2.5. Cardiac MRI

Last but not least, improvements in cardiac MRI protocols have made the imaging of microcirculation feasible by utilizing the diffusion of contrast medium from the microvasculature into the interstitium during the first pass. Integrated approaches, such as the Fermi model [28], have been formed to assess MBF which, together with microvascular perfusion resistance index, are good measures of CMVD [29,30]. Moreover, global CFR measurement is feasible via cardiac MRI, providing additional prognostic information [31]. Among cardiac MRI’s limitations, however, are the presence of imaging artifacts and the restricted use of gadolinium in chronic kidney disease patients.

### 2.6. Biomarkers

With regards to biomarkers, there have been several studies evaluating their role in CMVD (Table 1). Schroder et al. proceeded to an analysis of 92 biomarkers in women with CMVD and, even though they detected a component of six anti-inflammatory biomarkers that was associated with CMVD even after adjustment for known risk factors, it did not provide additional predictive value [32]. In this context, serum soluble CD40 ligand and high sensitivity C reactive protein (CRP) have been linked with CMVD, highlighting the inflammatory process mediating this entity [33,34,35]. Recently, Safdar et al. demonstrated the utility of renalase, a marker of endothelial function and inflammation, in patients with angina related to CMVD [36]. In the latest study of Suhrs et al., 17 inflammatory biomarkers were negatively correlated with coronary flow velocity reserve, further displayed the role of inflammation [37]. Despite their potential usefulness, validation in large-scale trials is warranted. Last but not least, microRNAs have been at the forefront in atherosclerosis research due to their potential as biomarkers as well as therapeutic approaches in ischemic diseases [38,39,40,41]. Several microRNAs (mir-125b, mir-181b, mir-200, mir-146) have been linked with the regulation of inflammatory processes and their use in the field of CMVD remains to be explored [42,43,44,45]. Last but not least, microRNAs have been at the forefront in atherosclerosis research due to their potential as biomarkers as well as therapeutic approaches in ischemic diseases [38,39,40,41]. Several microRNAs (mir-125b, mir-181b, mir-200, mir-146) have been linked with the regulation of inflammatory processes and their use in the field of CMVD remains to be explored [42,43,44,45].

## 3. The Role of Inflammation in the Pathogenesis of CMVD

### 3.1. Principal Pathophysiologic Mechanisms

Evidence from studies in autoimmune rheumatic diseases have stressed the decreased nitric oxide (NO) bioavailability together with the high assembly of reactive oxygen species (ROS) as the link between the pro-inflammatory state and endothelial dysfunction observed in CMVD [53]. CMVD can be maintained by a variety of physiological processes that result in either impaired dilatation or enhanced constriction of coronary microvessels [53]. Following endothelial dysfunction, the ensuing inflammation and immune system dysregulation are driving forces accelerating the atherosclerotic process, consequently affecting the microvasculature. Several chemokines and cytokines are involved, with interleukin IL-1, IL-6 and TNF-α being crucial mediators of the inflammatory cascade [54]. Systemic inflammation is further exacerbated by pro-inflammatory circulating microparticles, which are believed to be responsible for NO modulation and cytokine release as well as monocyte recruitment [55]. These modifications eventually impede myocardial blood flow ability to adjust to variations in myocardial oxygen demand. Impaired vasodilation can be a result of either non-modified risk factors such as diabetes, obesity, and hypertension or either endothelium–independent mechanisms, referring to the development of nitrate resistance due to decreased cyclic GMP production [56]. The exacerbation of secreting oxygen species in contribution with reduced NO bioavailability drive a chain reaction of signaling events that promotes heart fibrosis and myocyte stiffness [57].

The normal coronary physiology is disrupted to varied degrees as a result of CMVD. Patients with hypertrophic cardiomyopathy and those with arterial hypertension have structural abnormalities that are responsible for the development of CMVD [58]. In patients with non-obstructive coronary disease, dysfunction of microvasculature can be a result of several factors, such as increased heart rate, reduced diastolic time, decreased driving blood pressure, and left ventricular inotropism, which need to be considered when assessing microvascular function. Finally, the coronary blood flow linearly affected by the pulsative pattern of the heart and the intramyocardial and intraventricular pressures. As such, abnormalities in the diastolic phase of the cardiac cycle altered the myocardial perfusion [56,59].

### 3.2. Hypertension

**Figure 1:**
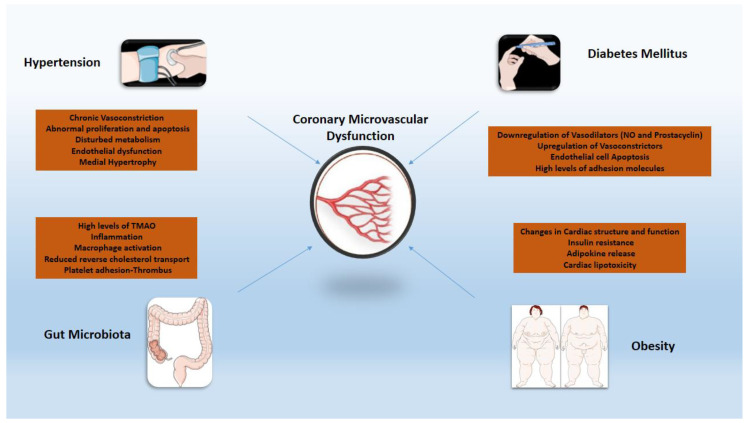
Inflammatory mechanisms of cardiovascular risk factors implicated in coronary microvascular dysfunction. TMAO: trimethylamine N-oxide; NO: nitric oxide.

Chronic low-grade systemic inflammation seen in the cluster of comorbidities (i.e., diabetes mellitus, obesity, arterial hypertension) that are considered risk factors for CMVD merits specific attention [60,61] (Figure 1). Arterial hypertension increases the likelihood of death from ischemic heart disease over time. In developed countries, at least 30% of individuals have a history of hypertension, and it is independently related to poor cardiac prognosis following an acute myocardial infarction (MI) [62]. Severe microvascular damage inside the infarct zone presents promptly as microvascular blockage and affects approximately half of all STEMI patients. In patients with persistent microvascular obstruction, progressive irreversible capillary degradation occurs, leading to infarct zone hemorrhage, which is an independent predictor of death or heart failure long-term [63,64]. The underlying pathophysiology includes inflammatory stimuli paired with increased activation of the renin–angiotensin–aldosterone system, resulting in endothelium-dependent CMVD by recruiting cell adhesion molecules [65]. Oxidative stress is also central as an orchestrator or collaborator of inflammation in hypertension-related vascular aging through mediators such as osteoprotegerin and the Sphingosine Kinase 1 gene [66,67]. The increased oxidative stress could further potentiate the pro-inflammatory effects of matrix metalloproteinases (MMPs), through the promotion of a secretory endothelial cell phenotype which aids vascular aging [68] and, consequently, CMVD. Patients with hypertrophic cardiomyopathy and arterial hypertension have structural abnormalities that are responsible for the development of CMVD [58]. The morphological alterations found in each of these disorders are defined by unfavorable remodeling of intramural coronary arterioles, resulting in medial wall thickening (mostly due to smooth muscle hypertrophy and increased collagen deposition) and varying degrees of intimal thickening [69].

### 3.3. Diabetes

In individuals with diabetes mellitus (DM), CMVD is often the prodrome of overt CAD and is a strong predictor of complications and mortality [70,71,72]. Inflammation in DM, induced by oxidative stress and the dysfunctional endothelium, plays a key role in the development of CMVD through the augmented expression of adhesion molecules and inflammatory cytokines as well as the mobilization of vascular smooth muscle cells [73,74]. Vascular adhesion protein-1 (VAP-1), another molecule involved in the pathophysiology of DM, is also overexpressed by damaged endothelial cells, affecting leukocyte recruitment and advanced glycation end products (AGE) generation, thus promoting a pro-inflammatory state [75,76,77]. Chemokines such as CC-chemokine ligand 2–3 and CXC-chemokine ligand 8 have also been found to be involved in leukocyte mobilization and macrophage recruitment, resulting in an augmented inflammatory response in the setting of DM [78]. TNF-α values correlated with the levels of glucose and cystatin C, confirming that diabetes plays an orchestrating role in the progress of CMVD [79]. Coronary microvascular dysfunction has an independent relationship with cardiac or all-cause mortality in people with and without diabetes, allowing for incremental risk classification. Patients with diabetes and impaired CFR have by far more increased risk for cardiac events than those with normal CFR [15]. Importantly, studies have stressed the importance of strict glycemic control in maintaining vascular integrity [74,75], a finding that could be partly justified by attenuation of the above mentioned pro-inflammatory, DM-induced effects via optimal management [80,81].

### 3.4. Obesity

Weight status constitutes a significant role in the progression of atherosclerotic disease [82]. Obesity is characterized by excessive expansion of visceral white adipose tissue mass, also known as adiposopathy. Adiposopathy is comprised of adipocyte hypertrophy, decreased adipose tissue blood flow, altered oxygen levels within the tissue, a state of chronic low-grade inflammation and blunted lipid metabolism [83,84,85]. The latter includes impaired capacity for storing the surplus of dietary lipids, resulting in deposition of ectopic fat accumulating in body locations where it is not physiologically stored, such as the liver and muscle, and a shift to visceral adipose tissue (fat storage in the intraperitoneal and retroperitoneal spaces), contributing to increased circulating free fatty acids, oxidative stress, systemic inflammation, adipokine dysregulation and insulin resistance [83,84,85,86,87,88]. However, epicardial adipose tissue has several unique properties that distinguish it from other depots of visceral fat due to the common microcirculation of epicardial tissue and the underlying myocardium [89]. The accumulation of epicardial fat is closely associated with an impaired myocardial microcirculation, cardiac diastolic filling abnormalities, increased vascular stiffness, and left atrial dilatation in obese people [90,91]. More specifically, high levels of TNF-α induce the secretion of adhesion and chemoattractant molecules such as vascular cell adhesion molecule-1, intercellular adhesion molecule-1, and monocyte chemoattractant protein-1 while reducing nitric oxide availability. Resistin hormone stimulates the proliferation of smooth muscle cells and the over-secretion of endotelin-1, leading to endothelial dysfunction [92]. Obesity-related reductions in myocardial blood flow due to CMVD, combined with increased cardiac metabolic demand due to increases in ventricular mass, volume expansion, higher filling pressures, and greater cardiac output, may create a perfect background for the occurrence of myocardial oxygen supply–demand mismatch [15]. Finally, an unfavorable correlation was observed between CFR and increased LDL cholesterol where treatment with pioglitazone and lipid-lowering therapy seemed to improve CFR [93].

### 3.5. Gut Microbiota

This term describes the various commensal microbial species in the gastrointestinal tract [94]. During the last decade, several studies reported the potential association between gut microbiota and atherosclerosis [94,95]. Choline, betaine and L-carnitine are metabolized to trimethylamine (TMA) which is generated to trimethylamine N-oxide (TMAO), a gut microbe-dependent metabolite [94,95]. Studies showed that increased TMAO level induced the activation of NF-kappa B (NF-κB) pathway and increased the expression of pro-inflammatory genes including inflammatory cytokines, adhesion molecules and chemokines [96]. Oxidative stress and NOD-like receptor protein 3 (NLRP3) inflammasome activation could also be triggered by TMAO and inflammatory cytokines such as IL-18 and IL-1β released. As such, diet has an important role in affecting the concentration of TMAO levels and the progression of atherosclerosis [97,98]. Latest studies showed that the use of broad-spectrum antibiotics for 3 to 4 weeks suppressed TMAO levels, ameliorating age-related oxidative stress and arterial dysfunction in mice [99]. The ability of CAD patients’ microbiota to generate ‘secondary’ bile acids enhanced the variety of the bile acid pool in both feces and serum. Under a high fat diet, this mechanism impeded hepatic bile acid synthesis and resulted in higher blood cholesterol levels. The CAD microbiota enhanced circulatory lipopolysaccharides levels as well as pro-inflammatory cytokine expression, while activating intestinal and systemic T help responses and decreasing Treg cell dispersion [100]. Finally, the use of probiotics, synbiotics, and probiotic functional products in a study of 90 obese patients with CAD proved beneficial, controlling plasma TMAO and HDL-C levels [101]. Further studies are needed to evaluate the efficacy of inhibiting various steps of TMAO production for the management atherosclerotic disease.

## 4. Anti-Inflammatory Drugs

**Figure 2:**
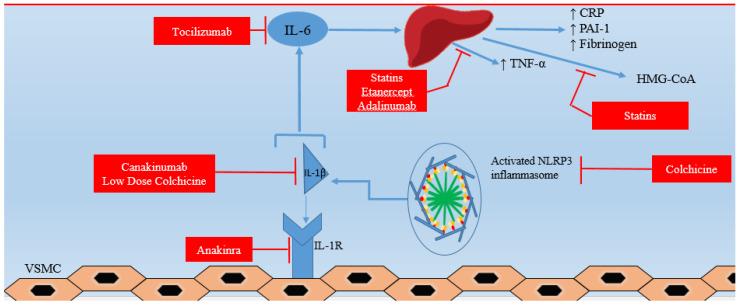
Anti-inflammatory approaches in cardiovascular diseases. IL: interleukin; NLRP3: NOD-like receptor protein 3; TNF: tumor necrosis factor; CRP: C reactive protein; PAI: plasminogen activator inhibitor; VSMC: Vascular Smooth Muscle Cell; HMG-CoA: 3-hydroxy-3-methylglutaryl-CoA.

Several anti-inflammatory drugs have been assessed over the years in either small or large randomized controlled trials (Table 2). Their aim was to reduce the inflammatory burden of coronary arteries, reducing the secretion of inflammatory cytokines while improving atherosclerotic plaque stabilization. Anti-inflammatory medications could constitute possible therapeutic options in the management of CMVD (Figure 2).

### 4.1. Statins-Aspirin

The effects of statins are well known for their pleiotropic actions and they have been the cornerstone of treatment for cardiovascular risk reduction and prevention [107]. Statins inhibit the critical step of cholesterol synthesis in which 3-hydroxy-3-methylglutaryl coenzyme A (HMG-CoA) is transformed to mevalonate by the enzyme HMG-CoA reductase [107]. Statins have been proven to reduce serum cholesterol along with a significant reduction in the morbidity and mortality of cardiovascular disease [107]. Statins act as anti-inflammatory agents, since the mevalonate pathway also influences endothelial function, inflammatory response, and coagulation [108]. The suppression of pro-inflammatory cytokines (TNF-α) expression paired with the upregulation of anti-inflammatory cytokines (IL-10) has been observed in animal models [109,110]. The largest to date JUPITER trial showed a significant reduction of major adverse cardiovascular events (MACE) associated with lower high sensitive C-reactive protein (hsCRP) levels, in the group treated with rosuvastatin rather than placebo [111]. Moreover, statins seem to stabilize the atherosclerotic plaque with thickened fibrous caps and macrocalcification via a reduction in lipid content [108,112].

Luo et al. showed that statin treatment was associated with low morbidity and mortality in patients with coronary microvascular dysfunction, during a follow up of 5 years. That was strengthened when the results were adjusted for various factors of the disease, such as smoking, sex, hyperhomocysteinemia, abnormal glucose tolerance, diabetes, hypertension and hyperlipidemia [113]. Several studies have evaluated the use of statins with regard to CMVD. Rosuvastatin was efficacious in improving CFR in hypertensive patients after 12 months of treatment [114,115,116], while the use of statin after an acute myocardial infarction resulted in an increase of myocardial flow reserve at 2 weeks, imaged via cardiac PET [117]. Meta-analytic results provided further confirmation of the beneficial effect of statins on CMVD in patients without significant epicardial stenosis, by means of improved CFR [118]. Although there is currently no recommendation for the consistent introduction of statins in CMVD patients, these with high CV risk are candidates for early statin prescription.

In this aspect, aspirin increases cell apoptotic rate by decreasing the cell proliferation rate of vascular smooth muscle cells. It suppresses atherosclerosis progression by down-regulating the expression of NFκB1 and its targets, which might well provide us with more therapeutic strategies for treatment of the disease [119]. There is no convincing evidence that low-dose aspirin is beneficial in these people. Aspirin may reduce microvascular thrombotic events in individuals with atherosclerotic-related comorbidities [120].

### 4.2. Colchicine

(NOD)-like receptor protein 3 (NLRP3) inflammasome is a protein complex with an important role in inflammatory progression [6]. Cholesterol crystals are present through all stages of atherosclerosis and can activate the NLRP3 inflammasome within these inflammatory cells to produce IL-1β and IL-18, key mediators in the inflammatory cascade that drive plaque progression and instability [6]. Colchicine has been studied as an antagonist of NLRP3 inflammasome and, consequently, of IL-1β production. Firstly, a cohort study included 64 patients with stable CAD and abnormal hsCRP levels, notes a significant drop of hsCRP in the colchicine treatment group [121]. The COLchicine Cardiovascular Outcomes Trial (COLCOT) strengthened the beneficial effects of early initiation of colchicine after myocardial infarction, decreasing the rates of cardiovascular death, resuscitated cardiac arrest, MI, stroke, or urgent hospitalization for angina requiring coronary revascularization [103,122].

There is only one study referring to CMVD which revealed that inhibition of inflammatory cytokines by colchicine or a receptor antagonist seems to improve CFR, myocardial contractility and relaxation in patients with rheumatoid arthritis [107].

### 4.3. Anakinra–Canakinumab

Anakinra is an IL-1 receptor antagonist, which was first used for rheumatoid arthritis treatment. It binds to the IL-1 receptor to exert powerful IL-1α and IL-1β blocking activity [123]. It has been found to downregulate CRP and IL-6 in patients with myocardial infarction. Several studies have been found to improve the microvascular dysfunction with potential benefits in ischemic stroke, hemorrhagic stroke, diabetes and CAD [5,124]. A double-blind, crossover, placebo-controlled trial of 23 patients with rheumatoid arthritis showed that administration of IL-1ra (Anakinra) caused a greater reduction of oxidative stress and IL-6. In addition, normalization of CRP levels as well as CFR and left ventricular function was observed after administration of Anakinra, in a mean follow up of 30 days [104]. Ikonomidis et al. performed a randomized trial and noted that anakinra resulted in a 29% improvement of CFR and an 18.7% improvement in global longitudinal strain after 3 months of treatment [125].

The CANTOS trial enrolled approximately 10,000 patients with a history of myocardial infarction and elevated hsCRP levels. Patients were stratified in canakinumab group and placebo, for a median follow up of 3.7 years. The CANTOS study showed that Canakinumab, a monoclonal antibody inhibitor of IL-1β, decreased the risk of cardiovascular events by lowering systemic inflammation in high cardiovascular risk patients [124,126]. More specifically, decrease of hsCRP and IL-6 was dose-dependent, while canakinumab at 150–300 mg every 3 months was found to reduce MACE by approximately 15% [5]. As such, further studies are needed for clarification of the utility of canakinumab in patients with CMVD, due to beneficial role in the whole progress of atherosclerosis.

### 4.4. Tocilizumab

Tocilizumab is a monoclonal antibody that competitively inhibits IL-6 receptor and has been shown to be useful in the treatment of rheumatoid arthritis, a chronic inflammatory disease [123]. Tocilizumab could improve endothelial function and decrease aortic stiffness in atherosclerotic patients [127,128,129]. In addition, CRP and fibrinogen were reduced in patients receiving tocilizumab [127,128,129]. On the other hand, the use of tocilizumab for atherosclerosis treatment is still debated since a recent study showed that patients receiving Tocilizumab had a mean increase in low-density lipoprotein (LDL) of 17 mg/dL, whereas another study found a 22% increase in LDL and total cholesterol and a 48% increase in fasting triglycerides in such patients [130].

Holte et al. showed that administration of Tocilizumab after MI did not affect CFR during hospitalization or after 6 months. Vascular cell adhesion molecule 1 (VCAM-1) levels were increased, but this was not associated with reduced CFR in these patients [105]. On the contrary, the treatment of rheumatoid arthritis patients with tocilizumab resulted in the improvement of CFR by 13% [125]. In a recently reported randomized controlled trial, the administration of tocilizumab 280 mg in patients with ST elevation myocardial infarction led to less extensive microvascular obstruction in cardiac MRI, highlighting a potential benefit in coronary microvasculature [131].

### 4.5. Etanercept-Adalinumab

Another target is TNF, whose inhibitors are available and well-evaluated drugs without severe adverse events. Agents from this drug category have been evaluated solely in patients with rheumatological conditions, additionally examining the incidence of MACE. In that subgroup a significant decrease in MACE was observed [132,133]. The ENTRACTE study showed that the TNF-α antagonists, Etanercept and Adalimumab, exert beneficial effects on atherosclerosis, blunting the progression of the subclinical disease via decreasing ICAM-1 and asymmetric dimethylarginine levels [134,135]. Furthermore, disruption of TNF-α signaling prevented angiotensin II-induced hypertension and aortic O_2_, while a reduction in resistance artery myogenic responsiveness was observed in mice [136,137].

With regards to CMVD, the treatment of psoriasis patients with etanercept for 4 months led to an 11% improvement in coronary flow reserve as reported in a randomized trial by Ikonomidis et al. [138], confirming the results of a previous prospective cohort study of 37 patients with psoriasis treated with TNF-α inhibitors by Piaserico et al. [139]. In the latter, the authors found that the improvement in CFR was correlated with the degree of reduction of inflammatory biomarkers [139]. In another study, twenty obese patients with type 2 diabetes were randomized to etanercept treatment (25 mg subcutaneously twice weekly for 4 weeks). Plasma levels of C-reactive protein and interleukin-6 decreased while microvascular vasodilatory responses remain unchanged. Due to the small sample studied, further evaluation of Etanercept is needed in larger cohorts [112,140].

## 5. Conclusions

Coronary microvascular dysfunction, a frequently encountered phenomenon in cardiology practice, is the result of endothelial dysfunction while systemic inflammation appears to be a significant contributing factor stemming from the cluster of comorbidities which are present. Recent pharmacological advances in the field of anti-inflammatory therapy may become essential for attenuating the residual cardiovascular risk in CAD with microvascular dysfunction.

## Figures and Tables

**Table 1:** Examined biomarkers in the field of coronary microvascular dysfunction.

Study	Biomarker	Outcome
Aslan et al. [33]	sCD40-L	sCD40-L was related to MVD in regression analysis
Dollard et al. [34]	hsCRP	hsCRP levels are proportional to disease severity
Safdar et al. [36]	Renalase	Independent predictor of coronary MVD even after adjustment for risk factors
Liang et al. [46]	VCAM-1	VCAM-1 is a significant factor differentiating obstructive CAD with CSX
Efe et al. [47]	Endocan	Endocan levels ≥2072 ng/L had a 72% sensitivity and 54% specificity for accurate prediction of CSX
Prasad et al. [48]	Uric acid	Uric acid was associated with markers of inflammation and coronary endothelial dysfunction in postmenopausal women
Altiparmak et al. [49]	Thiol	Specificity 84% and sensitivity 86% of CSX prediction with total thiol values ≤338.4 µmol/L
Schroder et al. [32]	Component of: CCL16 CXCL16 PGLYRP1 TNFR1 GDF15 TNFRSF10C	The 9-biomarker component was associated with MVD but did not provide further diagnostic utility
Mekonnen et al. [50]	suPAR	Association of suPAR with CFR
Bozcali et al. [51]	Galectin 3	↑ Galectin-3 in patients with CSX even after adjustment for risk factors
Tenekecioglou et al. [52]	HDL-C	↓ HDL-C is associated with systemic inflammation in CSX

sCD40-L: soluble CD40 ligand, MVD: microvascular dysfunction, hsCRP: high sensitivity C reactive protein, VCAM-1: vascular cell adhesion molecule-1, CAD: coronary artery disease, CSX: coronary syndrome X, CCL16: Chemokine (C-C motif) ligand 16, CXCL16: Chemokine (C-X-C motif) ligand 16, PGLYRP1: Peptidoglycan Recognition Protein 1, TNFR1: tumor necrosis factor receptor 1, GDF15: growth/differentiation factor 15, TNFRSF10C: TNF Receptor Superfamily Member 10c, suPAR: soluble urokinase-type plasminogen activator receptor, CFR: coronary flow reserve, HDL-C: high density lipoprotein cholesterol. ↑ indicates increased expression, ↓ indicates decreased expression.

**Table 2:** Therapeutic anti-Inflammatory agents.

DRUG	ACTION	TRIAL
**Aspirin**	Inhibitor of COX	Coronary Microvascular Angina Trial (CorMicA) [102]
**Colchicine**	Inhibition of NLRP3 inflammasome	COLCOT Trial [103]
**Anakinra**	Monoclonal antibody against IL-1 Receptor	Ikonomidis et al. [104]
**Canakinumab**	monoclonal antibody against IL-1-beta	CANTOS Trial [5]
**Tocilizumab**	IL-6 Receptor Inhibitor	Holte et al. [105]
**Etanercept**	TNF-α antagonists	ENTRACTE Trial [106]
**Adalimumab**	TNF-α antagonists	ENTRACTE Trial [106]

IL: interleukin; TNF: tumor necrosis factor; NLRP3: NOD-like receptor protein 3; COX: cyclooxygenase.

## Data Availability

Not applicable.
